# *LILRA6* copy number variation correlates with susceptibility to atopic dermatitis

**DOI:** 10.1007/s00251-016-0924-z

**Published:** 2016-06-22

**Authors:** M. R. López-Álvarez, W. Jiang, D. C. Jones, J. Jayaraman, C . Johnson, W. O. Cookson, M. F. Moffatt, J. Trowsdale, J. A. Traherne

**Affiliations:** 1 Immunology Division, Department of Pathology, University of Cambridge, Tennis Court Road, Cambridge, CB2 1QP UK; 2 Cambridge Institute for Medical Research, University of Cambridge, Cambridge Biomedical Campus, Wellcome Trust/MRC Building, Hills Road, Cambridge, CB2 0XY UK; 3 Molecular Genetics and Genomics Section, National Heart and Lung Institute, Imperial College London, Dovehouse Street, London, SW3 6LY UK

**Keywords:** LILR, Atopic dermatitis, AD, CNV

## Abstract

Leukocyte immunoglobulin-like receptors (LILR) are expressed mostly on myelomonocytic cells where they are mediators of immunological tolerance. Two LILR genes, *LILRA3* and *LILRA6*, exhibit marked copy number variation. We assessed the contribution of these genes to atopic dermatitis (AD) by analysing transmission in 378 AD families. The data indicated that copies of *LILRA6* were over-transmitted to affected patients. They are consistent with a contribution of LILR genes to AD. They could affect the equilibrium between activating and inhibitory signals in the immune response.

## Introduction

Atopic dermatitis (AD) is a chronic disease that affects around 10–20 % of children in western countries, characterised by pruritus and skin inflammation and frequently associated with food allergies and asthma (Werfel et al., 2014).

There are several factors that contribute to the development of AD such as environmental conditions (Zutavern et al., 2005; Roduit et al., 2011; Silverberg et al., 2011; Caroline et al., 2012), dysfunction of the skin barrier (Cork et al., 2009; Agrawal and Woodfolk, 2014) and viral and bacterial skin infections (Cho et al., 2001). AD can be developed when there is an abnormal or sustained activation of the skin innate immune system (McGirt and Beck, 2006; Maintz and Novak, 2011; Kasraie and Werfel, 2013, Kuo et al., 2013). Additionally, genetic factors have been associated to the development and vertical transmission of AD (Söderhäll et al., 2007; Irvine et al., 2011). Alterations in the function and signalling pathways and polymorphisms in Toll-like receptor 2 (TLR2) have been associated with the development of AD (Potaczek et al., 2011; Yu et al., 2015) whilst Killer immunoglobulin-like receptor (KIR), specifically KIR2DS1, seems to be associated with protection from AD (Niepiekło-Miniewska et al., 2013).

Leukocyte immunoglobulin-like receptors (LILR) are also innate immune receptors encoded, like KIR, within the leukocyte receptor complex (LRC) on chromosome 19q13.4 (Barrow and Trowsdale, 2008). *LILR* display limited genetic diversity (Young et al., 2001) apart from *LILRB3* and *LILRA6*, which exhibit considerable sequence polymorphism in the extracellular domains and *LILRA3* and *LILRA6* which also show variation in the number of copies (Torkar et al., 2000; Sudmant et al., 2010; Bashirova et al., 2014; Lopez-Alvarez et al., 2014). LILR can regulate the activity of TLR (Brown et al. 2004, Cao et al., 2009) and mediate the immunological tolerance (Manavalan et al., 2003; Kim-Schulze et al., 2006; Anderson and Allen, 2009). Currently, there is no data about the role of LILRB3 and LILRA6, although their recently discovered interaction with a cytokeratin 8-associated ligand suggests they could shape local inflammatory responses to epithelial tumours (Jones et al., 2016). LILRA3 has been described as a soluble receptor that binds HLA class I molecules and it has been associated with multiple sclerosis (An et al., 2016), systemic lupus erythematosus and Sjögren’s syndrome (Du et al., 2014).

Despite the strong genetic component together with the implication of the innate immune response in AD, in particular TLR and KIR receptors, there is no data available considering the association of LILR genes with AD. This is a preliminary study which tries to determine the impact of the variability of *LILRA3* and *LILRA6* loci among members of 378 AD families.

DNA samples, obtained from the UK DNA Banking Network (DBN), consisted of 1482 Caucasoid individuals from 378 families. Three hundred fifty-eight children had active AD. The children’s mean age was 10 years (±4 years). One hundred ninety-six (55 %) were male subjects (Sandford et al., 1995; Morar et al., 2006). Children lacking signs of disease were classified as unaffected. No significant association between sex or age and development of AD was found (*p* > 0.05). All subjects were of initial European ancestry and gave their informed consent.

*LILRA3* and *LILRA6* copy number was determined by quantitative PCR on genomic DNA extracted using standard techniques. Forward and reverse primers and a dual-labelled probe were designed to specifically amplify *LILRA3* and *LILRA6* genes avoiding any allelic variation identified to date (Lopez-Alvarez et al., 2014). Sequences were analysed for specificity using the primerBLAST tool from the National Centre for Biotechnology Information (http://www.ncbi.nlm.nih.gov/tools/primer-blast). In addition, all reactions contained specific primers and a probe for the *STAT6* gene, which has two copies per diploid human genome, and was used as an endogenous reference gene. All reactions were performed in quadruplicate for each sample to increase the accuracy of copy number scoring. Positive controls for each copy number type from IHW cell lines (Lopez-Alvarez et al., 2014) and a negative non-template control were included on each microtiter plate.

A total of 10 ng of genomic DNA was amplified under the following PCR conditions: 5 min at 95 °C, followed by 40 cycles of 95 °C for 15 s and 66 °C for 50 s, followed by 10 s at 40 °C, using the LightCycler 480 System (Roche Diagnostics Ltd., Burgess Hill, UK). *LILR* copy number was determined by a quantitative PCR comparative Ct method (Schmittgen and Livak, 2008).

Family-based association analysis was carried out using the transmission disequilibrium test (TDT) to examine the transmission rates for the number of copies of *LILRA3* and *LILRA6* from parents to affected offspring. The analysis was performed using Unphased software (Dudbridge, 2008) which implements maximum-likelihood inference on genotype effects. In addition, FBAT software version V2.04 was used to perform the association analyses using the additive model and bi-allelic mode (data not shown).

First of all, we determined the variation in the number of copies of *LILRA3* and *LILRA6* loci in the cohort of families. The number of copies of *LILRA3* per haplotype varied from 0 to 2 copies, whilst *LILRA6* showed between 0 and 4 copies. One copy was the most frequent for *LILRA3* and *LILRA6* with frequencies over 70 % in both cases (Table 1). The frequencies obtained per diploid genome matched very closely to those previously reported (data not shown, Lopez-Alvarez et al., 2014).

**Table 1:** Frequencies of the number of copies in *LILRA3* and *LILRA6* per haplotype

Marker	Number of copies	Total	AD		*P* value
			Unaffected	Affected	
*LILRA3*	0	613 (20.7)	401 (20.9)	212 (20.3)	0.714
	1	2339 (78.9)	1512 (78.7)	827 (79.4)	
	2	12 (0.4)	9 (0.5)	3 (0.1)	
*LILRA6*	0	217 (7.3)	143 (7.4)	74 (7.1)	0.948
	1	2107 (71.2)	1370 (71.3)	737 (70.7)	
	2	617 (20.8)	395 (20.6)	222 (21.3)	
	3	19 (0.6)	12 (0.6)	7 (0.7)	
	4	4 (0.1)	2 (0.1)	2 (0.1)	

Number of copies denotes copies per haplotype. Frequencies are given in the parentheses

Regarding the transmission of *LILRA3* and *LILRA6* among families, in relation to disease (Table 2), *LILRA6* showed a significant association with AD (*p* = 0.0025). There was a slight over-transmission of one copy of *LILRA6* to affected children (0.7197 vs. 0.6842, *p* = 0.002, OR 1.45, 95 % CI 1.14–1.85), suggesting that the presence of one copy of *LILRA6* on each chromosome could be a risk factor for AD. In contrast, haplotypes missing *LILRA6* (0 copies per haplotype) were under-transmitted (0.0636 vs. 0.0933). Analysis of the data with FBAT software (see Methods) also revealed the above associations (data not shown).

**Table 2:** TDT results for *LILRA3* and *LILRA6* in AD affected families

	Number of copies	Trans	Untrans	T-Freq	U-Freq	*P* value
*LILRA3*	0	157	432	0.2224	0.2006	0.980518
	1	547	1715	0.7748	0.7962	
	2	2	7	0.002833	0.00325	
*LILRA6*	0	44	200	0.06358	0.09325	0.00254137
	1	498	1467	0.7197	0.6842	
	2	145	462	0.2095	0.20155	
	3	4	14	0.00578	0.00653	
	4	1	1	0.001445	0.0004664	

Number of copies denotes copies of the gene on the same haplotype. Trans is the estimated count of this haplotype to affected offspring. Untrans is the estimated number of non-transmissions of this haplotype. TDT-like counts of transmitted and untransmitted allelic copies are not given as they cannot be accurately defined when parents are missing. T-Freq is the frequency of the haplotype in affected offspring. U-Freq is the frequency of the haplotype among untransmitted haplotypes. *P* value is the overall significance for the locus

In several families, the transmission of *LILRA3* and *LILRA6* genes was consistent with duplication of these genes on a chromosome. Twelve individuals (~1 %), distributed among five families, would have two copies of *LILRA3* on the same chromosome, being the only possible arrangement to explain the segregation through the next generation. The incidence of two copies of *LILRA6* on the same chromosome was more frequent (~7 %). In those individuals with a higher number of copies of *LILRA6*, the number of copies was not evenly distributed on each chromosome. For example, some possessed four copies on one chromosome and two on the other.

This is the first study considering the effect of LILR receptors on the development of AD. We analysed the variation in the number of copies of *LILRA3* and *LILRA6* genes in a series of a family trios suffering from the disease.

AD is characterised by a Th2-mediated response (Novak et al., 2003; Chu et al., 2011) triggered by dendritic cells (DC) (Moser and Murphy, 2000). LILRA3 is a soluble protein which seems to be an antagonist of inhibitory LILRs (Poon et al., 2005) and could also have an important role in establishing Th1 or Th2 responses (Thomas et al., 2010). The lack of LILRA3 could lead to the early conclusion of the antiviral defence, supporting chronic infection and favouring the development of autoimmune diseases, like multiple sclerosis and Sjögren’s syndrome (Pender, 2009; Thomas et al., 2010). Unlike in autoimmune diseases (Wiśniewski et al., 2013; Du et al., 2014), the number of copies of *LILRA3* was not significantly different between AD unaffected and affected individuals without considering their relatedness (Table 1) or in transmission frequencies of gene copies between unaffected and affected offspring.

LILRA6 is an activating receptor encoded by a polymorphic gene that shows variation in the number of copies (Bashirova et al., 2014; Lopez-Alvarez et al., 2014). Like *LILRA3*, there were no differences in the number of copies between unaffected and AD affected individuals but we observed significant differences in the transmission of the number of copies of *LILRA6* within families (Table 2). The results showed a modest over-transmission of one copy of *LILRA6* whilst haplotypes lacking this gene were under transmitted. These results suggest that the lack of *LILRA6* could be a protective factor against AD. Indeed, the activating nature of this receptor upon binding with the specific ligand could trigger the immune response and promote the development of atopic dermatitis. In contrast, two or more copies of *LILRA6* showed no influence on disease risk. The reason for this is unclear at present but it could mean that the homologues are disrupted genes or that they differ functionally.

*LILRA6* CNV may influence the level of the activating receptor on the cell surface, potentially affecting signalling upon LILRB3/A6 ligation (Bashirova et al., 2014). Thus, the variation observed here could affect the equilibrium between activating and inhibitory signals and the balance of the immune response. An alternative explanation could be that the effects we observe reflect linked genes such as KIR on NK cells. In this context, some HLA class I alleles, such as HLA-B*57:01 and HLA-B*44:02 which are ligands for KIR receptors, have been associated with AD in several GWAS studies (Hirota et al., 2012; Weidinger et al., 2013). Also, *KIR2DS1* may be a protective factor in AD, although its effect remains unclear (Kusnierczyk, 2013).

In conclusion, we have assessed the variation in the number of copies of *LILRA3* and *LILRA6* in a series of AD family trios. Among them, only the transmission of one copy of *LILRA6* within families was potentially related to the development of AD. Further studies should be done in order to clarify the role of LILRA6 and its possible ligands in the development of AD.

## References

[CR1] Agrawal R, Woodfolk JA (2014). Skin barrier defects in atopic dermatitis. Curr Allergy Asthma Rep.

[CR2] An H, Lim C, Guillemin GJ (2016). Serum leukocyte immunoglobulin-like receptor A3 (LILRA3) is increased in patients with multiple sclerosis and is a strong independent indicator of disease severity; 6.7kbp LILRA3 gene deletion is not associated with diseases susceptibility. PLoS One.

[CR3] Anderson KJ, Allen RL (2009). Regulation of T cell immunity by leukocyte immunoglobulin-like receptors: innate immune receptors for self antigen-presenting cells. Immunology.

[CR4] Barrow AD, Trowsdale J (2008). The extended human leukocyte receptor complex: diverse ways of modulating immune responses. Immunol Rev.

[CR5] Bashirova AA, Apps R, Vince N (2014). Diversity of the human LILRB3/A6 locus encoding a myeloid inhibitory and activating receptor pair. Immunogenetics.

[CR6] Brown D, Trowsdale J, Allen R (2004). The LILR family: modulators of innate and adaptive immune pathways in health and disease. Tissue Antigens.

[CR7] Cao W, Bover L, Cho M (2009). Regulation of TLR7/9 responses in plasmacytoid dendritic cells by BST2 and ILT7 receptor interaction. J Exp Med.

[CR8] Caroline R, Frei R, Loss G (2012). Development of atopic dermatitis according to age of onset and association with early-life exposures. J Allergy Clin Immunol.

[CR9] Cho SH, Strickland I, Boguniewicz M (2001). Fibronectin and fibrinogen contribute to the enhanced binding of *Staphylococcus aureus* to atopic skin. J Allergy Clin Immunol.

[CR10] Chu CC, Di Meglio P, Nestle FO (2011). Harnessing dendritic cells in inflammatory skin diseases. Semin Immunol.

[CR11] Cork MJ, Danby SG, Vasilopoulos Y (2009). Epidermal barrier dysfunction in atopic dermatitis. J Investig Dermatol.

[CR12] Du Y, Su Y, He J (2014). Impact of the leukocyte immunoglobulin-like receptor A3 (LILRA3) on susceptibility and subphenotypes of systemic lupus erythematosus and Sjögren’s syndrome. Ann Rheum Dis.

[CR13] Dudbridge F (2008). Likelihood-based association analysis for nuclear families and unrelated subjects with missing genotype data. Hum Hered.

[CR14] Hirota T, Atsushi T, Kubo M (2012). Genome-wide association study identifies eight new susceptibility loci for atopic dermatitis in the Japanese population. Nat Genet.

[CR15] Irvine AD, McLean WH, Leung DY (2011). Filaggrin mutations associated with skin and allergic diseases. N Engl J Med.

[CR16] Jones DC, Hewitt CR, López-Álvarez MR (2016). Allele-specific recognition by LILRB3 and LILRA6 of a cytokeratin 8-associated ligand on necrotic glandular epithelial cells. Oncotarget.

[CR17] Kasraie S, Werfel T (2013) Role of macrophages in the pathogenesis of atopic dermatitis. Mediat Inflamm. Article ID 942375 10.1155/2013/942375 PMC3603294 23533313

[CR18] Kim-Schulze S, Scotto L, Vlad G (2006). Recombinant Ig-like transcript 3-Fc modulates T cell responses via induction of Th anergy and differentiation of CD8^+^ T suppressor cells. J Immunol.

[CR19] Kuo IH, Yoshida T, De Benedetto A (2013). The cutaneous innate immune response in patients with atopic dermatitis. J Allergy Clin Immunol.

[CR20] Kusnierczyk P (2013). Killer cell immunoglobulin-like receptor gene associations with autoimmune and allergic diseases, recurrent spontaneous abortion, and neoplasms. Front Immunol.

[CR21] Lopez-Alvarez MR, Jones DC, Jiang W (2014). Copy number and nucleotide variation of the LILR family of myelomonocytic cell activating and inhibitory receptors. Immunogenetics.

[CR22] Maintz L, Novak N (2011). Modifications of the innate immune system in atopic dermatitis. J Innate Immun.

[CR23] Manavalan JS, Rossi PC, Vlad G (2003). High expression of ILT3 and ILT4 is a general feature of tolerogenic dendritic cells. Transpl Immunol.

[CR24] McGirt LY, Beck LA (2006). Innate immune defects in atopic dermatitis. J Allergy Clin Immunol.

[CR25] Morar N, Bowcock AM, Harper JI (2006). Investigation of the chromosome 17q25 PSORS2 locus in atopic dermatitis. J Investig Dermatol.

[CR26] Moser M, Murphy KM (2000). Dendritic cell regulation of T_H_1-T_H_2 development. Nat Immunol.

[CR27] Niepiekło-Miniewska W, Majorczyk E, Matusiak L (2013). Protective effect of the KIR2DS1 gene in atopic dermatitis. Gene.

[CR28] Novak N, Bieber T, Leung DYM (2003). Immune mechanisms leading to atopic dermatitis. J Allergy Clin Immunol.

[CR29] Pender MP (2009). Preventing and curing multiple sclerosis by controlling Epstein–Barr virus infection. Autoimmun Rev.

[CR30] Poon K, Montamat-Sicotte D, Cumberbatch N (2005). Expression of leukocyte immunoglobulin-like receptors and natural killer receptors on virus-specific CD8^+^ T cells during the evolution of Epstein-Barr virus-specific immune responses in vivo. Viral Immunol.

[CR31] Potaczek DP, Nastalek M, Okumura K (2011). An association of TLR2-16934A > T polymorphism and severity/phenotype of atopic dermatitis. J Eur Acad Dermatol Venereol.

[CR32] Roduit C, Wohlgensinger J, Frei R (2011). Prenatal animal contact and gene expression of innate immunity receptors at birth are associated with atopic dermatitis. J Allergy Clin Immunol.

[CR33] Sandford AJ, Moffatt MF, Daniels SE (1995). A genetic map of chromosome 11q, including the atopy locus. Eur J Hum Genet.

[CR34] Schmittgen TD, Livak KJ (2008). Analyzing real-time PCR data by the comparative C(T) method. Nat Protoc.

[CR35] Silverberg JI, Kleiman E, Lev-Tov H (2011). Association between obesity and atopic dermatitis in childhood: a case control study. J Allergy Clin Immunol.

[CR36] Söderhäll C, Marenholz I, Kerscher T (2007). Variants in a novel epidermal collagen gene (COL29A1) are associated with atopic dermatitis. PLoS Biol.

[CR37] Sudmant PH, Kitzman JO, Antonacci F (2010). Diversity of human copy number variation and multicopy genes. Science.

[CR38] Thomas R, Matthias T, Witte T (2010). Leukocyte immunoglobulin-like receptors as new players in autoimmunity. Clin Rev Allergy Immunol.

[CR39] Torkar M, Haude A, Milne S (2000). Arrangement of the ILT gene cluster: a common null allele of the ILT6 gene results from a 6.7-kbp deletion. Eur J Immunol.

[CR40] Weidinger S, Willis-Owen SAG, Kamatani Y (2013). A genome-wide association study of atopic dermatitis identifies loci with overlapping effects on asthma and psoriasis. Hum Mol Genet.

[CR41] Werfel T, Schwerk N, Hansen G (2014). The diagnosis and graded therapy of atopic dermatitis. Dtsch Arztebl Int.

[CR42] Young NT, Canavez F, Uhrberg M (2001). Conserved organization of the ILT/LIR gene family within the polymorphic human leukocyte receptor complex. Immunogenetics.

[CR43] Yu Y, Zhang Y, Zhang J (2015). Impaired Toll-like receptor-2-mediated Th1 and Th17/22 cytokines secretion in human peripheral blood mononuclear cells from patients with atopic dermatitis. Transl Med.

[CR44] Zutavern A, Hirsch T, Leupold W (2005). Atopic dermatitis, extrinsic atopic dermatitis and the hygiene hypothesis: results from a cross-sectional study. Clin Exp Allergy.

